# Do not neglect calcium: a systematic review and meta-analysis (meta-regression) of its digestibility and utilisation in growing and finishing pigs

**DOI:** 10.1017/S0007114518000612

**Published:** 2018-04-03

**Authors:** Maciej M. Misiura, João A. N. Filipe, Carrie L. Walk, Ilias Kyriazakis

**Affiliations:** 1 Agriculture, School of Natural and Environmental Sciences, Newcastle University, Newcastle upon Tyne NE1 7RU, UK; 2 AB Vista, Marlborough SN8 4AB, UK

**Keywords:** Calcium, Phytase, Phosphorus, Digestibility, Pigs

## Abstract

Ca digestibility and utilisation in growing pigs are not well understood, and are usually neglected in diet formulation. This has implications not only for the accurate determination of its requirements but also for its interactions with other nutrients. A systematic review and meta-analysis (meta-regression) of published trials was carried out to quantify factors affecting Ca absorption and utilisation, and to derive an estimate of Ca endogenous excretion. The analysis was carried out on the data from forty studies, corresponding to 201 treatments performed on 1204 pigs. The results indicated that although Ca absorption and retention (g/kg of body weight per d) increased with increasing Ca intake (*P*<0·001), non-phytate-P intake (*P*<0·001) and exogenous phytase supplementation (*P*<0·001), these values decreased with increasing phytate-P intake (*P*<0·05). Interactions between exogenous phytase and Ca intake, indicating reduced efficacy of this enzyme (*P*<0·001), and between phytate-P intake and exogenous phytase, counteracting the direct negative effect of phytate-P (*P*<0·05) on Ca absorption and retention, were also detected. There were no effects of animal-related characteristics, such as pig genotype in Ca absorption and retention. The large amount of variance explained in Ca absorption (90 %) and retention (91 %) supported our choice of independent variables. Endogenous Ca losses obtained via linear regression were 239 mg/kg of DM intake (95 % CI 114, 364). These outcomes advance the current understanding of Ca digestibility and utilisation, and should contribute towards establishing requirements for digestible Ca. Consequently, pig diets will be more correctly formulated if digestible Ca values are used in estimating requirements for Ca.

Ca and P play important roles in bone mineralisation and development^(^ ^1^ ^)^, as well as in many non-skeletal physiological processes^(^ ^2^ ^,^ ^3^ ^)^. Although Ca and P are interdependent and interact in their absorption and utilisation^(^ ^2^ ^)^, historically, Ca has not generated as much research interest as P. This is because P is one of the most expensive components in pig diets^(^ ^4^ ^,^ ^5^ ^)^ owing to the low digestibility of plant dietary P, and the consequent need to add costly inorganic P supplements. From an environmental point of view, P supplies are limited and non-renewable^(^ ^6^ ^,^ ^7^ ^)^, and a low P feed conversion efficiency may lead to the high excretion of water-soluble P in manure, which causes water pollution in the form of eutrophication^(^ ^8^ ^–^ ^10^ ^)^. On the other hand, inorganic Ca supplementation is cheap, widely accessible^(^ ^11^ ^)^ and excessive Ca excretion does not explicitly cause any environmental concerns.

However, several authors have emphasised the significance of understanding Ca absorption, utilisation and excretion not only to improve present estimates of Ca requirements but also to optimise P utilisation^(^ ^12^ ^,^ ^13^ ^)^. To date, there has been limited research on the subject of Ca digestibility in growing and finishing pigs, and only recently experiments have established these values for several plant-based and inorganic sources of Ca^(^ ^14^ ^–^ ^17^ ^)^. This absence of information reflects the difficulty in the accurate determination of Ca requirements and to a certain extent of P utilisation. Moreover, some authors have attributed bone disorders during reproduction to our inability to adequately estimate Ca requirements during growth^(^ ^18^ ^)^. As a result, current National Research Council^(^ ^19^ ^)^ guidelines for Ca content in pig diets are expressed on the basis of total Ca, which ignore endogenous losses and the process of digestion. This fact often leads to over-supplementation of inorganic Ca, which promotes the formation of indigestible Ca–phytate-P (PP) complexes in the small intestine of pigs, reducing P digestibility^(^ ^20^ ^)^. Excess Ca was also shown to decrease protein digestibility by increasing gastric pH, albeit in broiler chickens^(^ ^21^ ^)^. Hence, a system of dietary recommendations based on digestible, rather than total, values of Ca would be expected to improve feed efficiency and reduce negative environmental impact caused by excessive P excretion, while ensuring that pig performance and health are maintained.

The objectives of this systematic review and meta-analysis (meta-regression) of previous digestibility trials were to (1) identify and quantify factors affecting Ca digestibility and utilisation, and (2) estimate the maintenance requirement for Ca, efficiency of Ca utilisation and its endogenous losses. The results of this study further enhance the current understanding of Ca digestion and metabolism in pigs, and should contribute towards the development of a dietary formulation system based on both digestible Ca and digestible P.

## Methods

Ethical approval was not required for this study, as the data were obtained from previous digestibility trials in which ethical approval has already been obtained by the trial investigators.

Throughout this paper, the terms meta-analysis and meta-regression are used interchangeably to describe the statistical methodology utilised in this study. Formally our analysis constitutes a meta-regression, which is a tool used in meta-analyses to examine the impact of moderator variables on study effect size using regression-based techniques.

### Search strategy

A review protocol (online Supplementary Material A) outlining strategies for systematic review and subsequent meta-analysis of literature on the subject of Ca digestibility and utilisation was developed first. Next, an initial scoping of the literature was carried out to determine the feasibility of this study^(^ ^22^ ^,^ ^23^ ^)^ and, consequently, multiple, full-scale literature searches were performed. The last literature search was performed on 4 January 2018.

The Web of Science and Scopus databases were selected to identify peer-reviewed articles. The literature searches were carried out in accordance with the review protocol using a combination of keywords outlined in Table 1. The grey literature was considered in three ways. First, Google Scholar and Science.gov were used to find any relevant data in non-peer-reviewed sources. Second, materials issued by major public bodies and agencies such as the European Food Safety Agency, along with publications by leading animal nutrition companies, were reviewed. Third, a small number of key authors and industry experts in the field were contacted personally.

**Table 1:** Outline of keyword searches used in the systematic review

Components	Keywords
Subject	∙ Calcium ∙ Calcium AND Phosp* ∙ Calcium AND Vitamin D ∙ Calcium AND Phytase
Response	∙ digest* OR utilisation OR utilization OR absor* OR metabol* OR require* OR level* OR concentration* ∙ ratio OR percent* OR rate OR proportion
Population	(pig NOT guinea pig) OR swine

Results of the literature searches were merged and exported into an EndNote library. It was necessary to filter these results and remove any duplicates, as the literature searches were not mutually exclusive. Afterwards, each paper was given its own unique accession number and considered for further analysis.

### Inclusion and exclusion criteria

The studies were eligible for inclusion in the present meta-analysis if they met the following criteria: (1) Ca and P balance data (mineral intake, faecal and urinal outputs, absorption and retention values) were presented simultaneously; (2) experiments were performed on growing (i.e. pigs that overcame stress associated with weaning) and finishing (50–100 kg body weight) pigs, irrespective of sex; (3) trials were carried out on breeds exhibiting capacity for lean tissue deposition favoured in modern commercial pig production systems; and (4) studies were published exclusively between 1990 and 2017, with older articles excluded to account for commercial husbandry and breeding changes. Experiments carried out on weaning piglets and sows were also excluded.

### Study selection

**Fig. 1:**
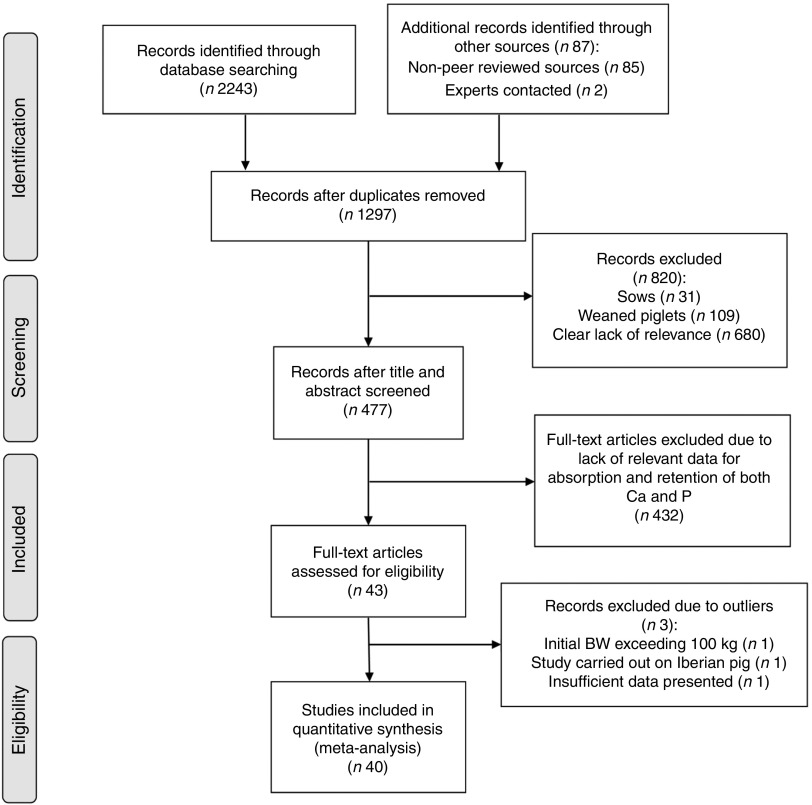
Study selection process. BW, body weight.

A total of 1297 unique records identified through the literature searches were examined using the aforementioned inclusion and exclusion criteria. These studies were assessed for their relevance in a three-stage process, largely based on Stewart *et al*.^(^ ^24^ ^)^. Initially, titles and abstracts were inspected by the primary reviewer and the studies deemed irrelevant were discarded. Next, a secondary reviewer was asked to go through a 25 % subsample of papers^(^ ^25^ ^)^ in order to calculate a kappa score^(^ ^26^ ^)^. A *κ* score quantifies the strength of agreement between reviewers and can be used to determine accuracy and reliability of the primary reviewer^(^ ^27^ ^)^. This assessment was passed in accordance with Pullin & Stewart^(^ ^28^ ^)^, as the *κ* score of 0·75 indicated substantial strength of agreement^(^ ^29^ ^)^. Subsequently, the remaining papers were read in full by the primary reviewer. In addition, the references in relevant articles generated by the literature searches were also screened as per Greenhalgh & Peacock^(^ ^30^ ^)^ guidelines. At this stage of the study selection, the main reason for rejection was lack of relevant data, especially for Ca digestibility and utilisation. A detailed summary of study selection procedures is presented in a Preferred Reporting Items for Systematic Reviews and Meta-Analyses (PRISMA) Flow Diagram^(^ ^31^ ^)^ in Fig. 1.

### Data extraction and critical appraisal

The following data, originating from thirty-nine peer-reviewed articles and one unpublished study, corresponding to 201 dietary treatments performed on 1204 pigs, were extracted into a purpose-built database in relation to the objectives: (1) study information (first author name, publication year and location), (2) dietary characteristics (diet type, main source of protein and energy, phytase presence and type, dietary Ca and P levels), (3) animal characteristics (breed and sex), (4) Ca and P balance studies (nutrient intake, faecal and urinal outputs, absorption and retention values) and (5) performance characteristics (initial and final body weight, average daily food intake). Each extracted data point corresponded to the observed mean of a treatment group. The aforementioned treatments were designed to study Ca digestibility and utilisation in animals fed variable Ca and P concentrations with and without additional exogenous phytase supplementation. A more detailed description of these dietary treatments can be found in ‘Study characteristics’ section below.

Reported sample sizes and standard errors were recorded in order to provide weights for the meta-analysis and to account for a variable degree of accuracy across studies^(^ ^32^ ^)^. This information was available in the majority (90 %) of articles. For the remaining four papers, in which standard errors or other measures of variability (such as 95 % CI, or standard deviations) were not given, estimated standard errors were derived and used as weights in accordance with the methodology of McPhee *et al*.^(^ ^33^ ^)^.

The data from the balance studies were used to calculate absorption and retention using the following definitions (adapted from Petersen & Stein^(^ ^34^ ^)^):1
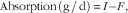

2
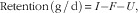
where *I* is the Ca intake in g/d (typically calculated as a dietary concentration of the mineral of interest (g/kg) multiplied by an average daily feed intake (g/d) throughout each experiment), *F* the faecal output of Ca in g/d and *U* the urinal output of Ca in g/d.

These calculated values were then compared against the figures for absorption and retention reported in these papers to minimise human error associated with data extraction and to check for omissions and internal consistency^(^ ^35^ ^)^. Furthermore, articles were critically appraised to quantify possible sources of bias that had the potential to affect the results of experiments using SYRCLE’s risk of bias tool^(^ ^36^ ^)^. This is one of the most comprehensive methods used for critical appraisal in animal studies, focusing on detection of selection, performance, detection, attrition and reporting biases and was adapted from Cochrane risk of bias tool for randomised controlled trials^(^ ^37^ ^)^. Results of the critical appraisal (online Supplementary Material B) indicated low risk of bias in the following four main categories (selection bias: sequence generation; selection bias: baseline characteristics; attrition bias; and reporting bias) and unknown or high risk in the remaining five main categories (selection bias: allocation concealment; performance bias: random housing; performance bias: blinding; detection bias: random outcome assessment; and detection bias: blinding). An additional source of bias was also identified and related to the nature of balance studies data. Methodological bias, owing to collection and measurement errors, which is likely to cause an over-estimation of absorption and retention values, was assumed to be present and constant for all selected studies.

### Overview of main variables and calculations

Absorption and retention defined by Equations (1) and (2) were chosen to quantify Ca digestibility and utilisation. However, these values are dependent on the amount of feed intake, which is conditional on the size of the pig. When animals are at the growing stage, feed intake is largely proportional to body weight and thus, as a first approximation to remove this dependency, Ca absorption and retention were scaled by the reported initial body weight. This specific approximation was used as information on the serial body weight was not available in any of the selected studies and the final body weight was only reported in twelve out of forty sources.

The main factors identified at the outset of this study, as potentially affecting Ca absorption and retention, were as follows: (1) total Ca intake (TCa) and the type of Ca source, (2) total P intake (TP) and its PP and non-PP (NPP) concentrations, (3) exogenous phytase intake (ExPhyt), (4) vitamin D intake, (5) pig sex and (6) pig genotype.

PP intake (g/d) was estimated using feed composition tables^(^ ^38^ ^)^. NPP intake (g/d) was calculated as the difference between the reported TP (g/d) and the estimated PP (g/d). Supplemented dietary exogenous phytase (phytase units (FTU)/kg) was multiplied by the average daily feed intake (g/d) to obtain daily ExPhyt (FTU/d). As for Ca absorption and retention, these observations were also scaled by the initial body weight.

The effects of different levels of vitamin D on Ca absorption and retention were explicitly investigated by only one study, with the remaining experiments supplying vitamin D at a constant level, either meeting or exceeding current National Research Council^(^ ^19^ ^)^ recommendations. Thus, this variable was not considered further in the analysis. Similarly, owing to insufficient data, it was not feasible to consider different Ca sources in the analysis, as dietary Ca levels were primarily derived from calcium carbonate.

Although there was diversity in pig genotypes among studies, it was possible to group them into three distinct classes in accordance with Averós *et al*.^(^ ^39^ ^)^ and Douglas *et al*.^(^ ^40^ ^)^. Group 1 contained Large White (LW) and Landrace pure breeds and their crosses, group 2 included Duroc pure breed and its crosses and group 3 combined commercial (synthetic) pig lines (offspring of various lines of gilts and boars originating mainly from the Pig Improvement Company). At this stage, one study was excluded as an outlier as it was carried out on Iberian pure breed pigs.

### Statistical analyses

#### Analysis of factors affecting calcium absorption and retention

As the data set was built from multiple experiments, heterogeneity was strongly suspected within the data set. To limit the possibility of obtaining biased parameter estimates in the meta-regression^(^ ^32^ ^)^, the existence of random study effects was formally assessed using likelihood ratio tests^(^ ^41^ ^)^. The goodness of fit between null models (that is, the intercept term only models, with either Ca absorption or retention as a dependent variable) and alternative, nested models with one added random effects term was compared using a *χ* ^2^ distribution with one degree of freedom. The results of these likelihood ratio tests provided strong evidence against null models. Hence, linear mixed effects regression (LMER) models^(^ ^32^ ^,^ ^41^ ^,^ ^42^ ^)^ were fitted to the data with either Ca absorption or retention as a dependent variable and a random effect associated with each study. The main fixed effects (i.e. covariates, or factors) were chosen from the *a priori* set of variables (TCa, PP, NPP, ExPhyt, pig sex, pig genotype) and, in addition, all possible two-way interactions between TCa, PP, NPP and ExPhyt were considered. Conditional *F*-tests were implemented to test the significance of the main fixed effects and their interactions^(^ ^43^ ^)^ at the 0·05 significance level. The incremental, manual stepwise backward–forward selection procedure was applied to select the final LMER model for each of the two dependent variables; non-significant factors were removed from the final LMER models. Furthermore, each observation was weighted by the inverse of a squared SEM to account for any potential heteroscedasticity originating from, for example, differences in sample sizes or different estimation procedures among studies included in the meta-analysis^(^ ^44^ ^)^. LMER model fitting was performed with the nlme package (version 3.1–131)^(^ ^45^ ^)^ in R (version 3.3.1)^(^ ^46^ ^)^ by using the restricted maximum likelihood method. Model validity was performed by examining QQ plots of the standardised residuals and scatterplots of the standardised residuals against the fitted values generated separately for the fixed and the random components of LMER models. These diagnostic plots did not reveal any major deviations from normality or heteroscedasticity of the fixed and the random effects residuals, and therefore did not invalidate the LMER model assumptions^(^ ^43^ ^)^. An example of diagnostic plots for the final Ca absorption LMER model is shown in the online Supplementary Material C. In addition, there were no considerable signs of multicollinearity between the main factors, as their correlations did not exceed 0·60^(^ ^39^ ^)^.

Quality of the final LMER models for Ca absorption and Ca retention was assessed by calculating the marginal *R* ^2^^(^ ^47^ ^)^ (*R* ^2^), the amount of variance explained by the fixed effects component of each model, using the MuMIn package (version 1.15.6)^(^ ^48^ ^)^.

In addition, an alternative data analysis was carried out by fitting an inverse variance weighted linear regression models with cluster robust variances^(^ ^49^ ^)^ (studies as clusters) to test whether the results are sensitive to methodological changes.

#### Estimation of endogenous and obligatory calcium losses

A secondary objective of the study was to derive estimates of endogenous Ca losses (i.e. the inevitable losses of Ca in the digestive tract) and obligatory Ca losses (i.e. a sum of the inevitable losses of Ca in the digestive tract and the inevitable losses of Ca excreted through the urine). These quantities can be estimated by using a factorial approach^(^ ^50^ ^)^, based on linear regression^(^ ^5^ ^)^, which involves extrapolation to the limit, where mineral intake is set to zero, with either Ca absorption (for an estimate of endogenous Ca losses) or Ca retention (for an estimate of obligatory Ca losses) chosen as a dependent variable. Consequently, this method was adapted for the present meta-analysis. LMER models with either Ca absorption or Ca retention as regressands, TCa, TP and ExPhyt as covariates, and a random effect associated with each study were fitted to the data. The *y*-intercept of these LMER models was then assumed to estimate either endogenous or obligatory Ca losses, depending on the choice of a response variable, and corresponds to an empirical scenario in which pigs are fed Ca- and P-free diets containing no additional phytase. Estimated losses could be adjusted to account for different dietary P and exogenous phytase levels.

These models, with fewer parameters than models with both PP and NPP covariates, were chosen to increase statistical power associated with estimates of the parameters of interest. The data analysis included values of TCa, which did not exceed National Research Council^(^ ^19^ ^)^ requirements for Ca to ensure that a linear relationship approximates both the intake–absorption and the intake–retention relationships. This range of data also reflects a more frequent and homogeneous sampling across studies included in the database.

As various units can be used to express endogenous excretion and obligatory losses, in this study the analysis was carried out 2-fold: (1) on the subset of the data satisfying the criteria outlined above, scaled by the body weight (*n* 174 out of 201 observations), and (2) on the subset of the data fulfilling the aforementioned requirement, scaled by the DM intake (DMI) (*n* 98 out of 201 observations).

#### Estimation of requirement for maintenance and gross efficiency of utilisation

The linear regression procedure^(^ ^5^ ^)^ used to estimate obligatory Ca losses can also be used to derive estimates for the maintenance requirement of Ca (defined as the amount of Ca that results in a zero Ca balance; that is, the Ca intake value, at which there is neither gain nor loss of Ca matter) and the gross efficiency of utilisation. The extrapolated *x*-intercept of this LMER model is the standard way of estimating the Ca requirement for maintenance (including any unavailable, undigested Ca). In addition, the gross efficiency of Ca utilisation can be obtained from an estimated value of the TCa parameter.

## Results

### Study characteristics

The list of thirty-nine selected peer-reviewed studies and one unpublished study is presented in the online Supplementary Material D. Data originated from three continents: twenty-four studies were carried out in North America, fourteen studies were carried out in Europe and two studies were carried out in Asia. The median publication year was 2011.

The treatments were designed to examine the response of Ca and P balances to Ca and P intakes ranging from 18·5 to 211·6 % of the current National Research Council^(^ ^19^ ^)^ guidelines for these two nutrients based on the weight of pigs in each trial. However, Ca and P intakes did not considerably exceed the National Research Council^(^ ^19^ ^)^ guidelines in over 86 % of these treatments (*n* 174 out of 201 observations). In addition, the majority of diets were calculated to meet or exceed the National Research Council^(^ ^19^ ^)^ recommendations for all other nutrients and such that the maintenance requirement for energy was exceeded by two to three times. The dietary treatments were predominantly formulated on the basis of total Ca:total P (thirty-one studies) as opposed to total Ca:digestible P ratio (nine studies). The ratio of total Ca:total P was kept constant for each dietary treatment within twenty-six studies.

Soyabean meal was the main source of protein in diets across experiments, with only eight studies opting for various combinations of rapeseed meal, potato protein, pea protein and egg white powder instead. However, primary sources of energy were more diverse with maize, barley and wheat reported. Dietary Ca was supplied through calcium carbonate either by itself (seventeen studies) or with small quantities of added monocalcium phosphate (seven studies) or dicalcium phosphate (fourteen studies); two studies did not report this information. Exogenous phytase was supplemented to dietary treatments in twenty-one studies predominantly through 3-phytase (nineteen studies), as opposed to 6-phytase (two studies).

The sample sizes in experiments ranged from two to twelve pigs per treatment group. In all, twenty seven studies performed experiments on barrows, seven studies on gilts, five studies on a mixture of gilts and barrows and one study on boars. The median adaptation period to the dietary treatments and the median length of faecal and urinal samples collection were both found to be 7 d. The faecal and urinal samples were generally collected either once or twice daily. Experiments included in the database were designed as either randomised block designs (77·5 % of studies) or Latin square designs (22·5 % of studies).

Descriptive statistics across studies for the main continuous (Table 2) and categorical (Table 3) variables used in the LMER modelling are shown below.

**Table 2:** Descriptive statistics for the main continuous variables included in the meta-analysis (Mean values and standard deviations; medians and minimum and maximum values)

Variables	*n*	Mean	sd	Median	Min	Max
Unscaled data						
Dependent variables						
Ca absorption (g/d)	201	6·00	2·33	5·99	0·29	13·50
Ca retention (g/d)	201	5·33	2·37	5·22	0·24	13·26
Independent variables						
TCa (g/d)	201	10·7	4·34	10·5	1·82	26·3
TP (g/d)	201	7·56	3·16	7·08	2·39	19·1
PP (g/d)	201	3·88	1·97	3·61	0·94	9·92
NPP (g/d)	201	3·68	2·08	3·13	0·76	14·4
ExPhyt (phytase units/d)	201	431	784	0·00	0·00	3858
Data scaled by the initial BW						
Dependent variables						
Ca absorption (g/kg of BW per d)	201	0·178	0·085	0·169	0·007	0·543
Ca retention (g/kg of BW per d)	201	0·158	0·080	0·153	0·006	0·533
Independent variables						
TCa (g/kg of BW per d)	201	0·313	0·154	0·271	0·046	1·04
TP (g/kg of BW per d)	201	0·222	0·113	0·183	0·074	0·794
PP (g/kg of BW per d)	201	0·110	0·066	0·091	0·032	0·499
NPP (g/kg of BW per d)	201	0·111	0·069	0·094	0·023	0·367
ExPhyt (phytase units/kg of BW per d)	201	10·8	20·3	0·00	0·00	99·2

TCa, total Ca intake; TP, total P intake; PP, phytate-P intake; NPP, non-phytate-P intake; ExPhyt, exogenous phytase intake; BW, body weight.

**Table 3:** Descriptive statistics for the main categorical variables included in the meta-analysis

Variables	*n*
Genotype	
Group 1 (LW, L, LW and L crosses)	107
Group 2 (D and D crosses)	49
Group 3 (commercial pig lines)	45
Sex	
Barrows	143
Gilts	30
Gilts and barrows	24
Boars	4

LW, Large White; L, Landrace; D, Duroc.

### Analysis of factors affecting calcium absorption

The mean Ca intake was 0·313 (se 0·154) g/kg of body weight (BW) per d and the mean Ca absorbed was estimated to be 0·178 (se 0·085) g/kg of BW per d (ranging from 0·007 to 0·543 g/kg of BW per d) (Table 2). The summary of the main significant fixed effects, together with the significant two-way interactions between the covariates in the final LMER model for Ca absorption, is presented in Table 4.

**Table 4:** Main significant fixed effects and their two-way interactions in the final linear mixed effects regression model for calcium absorption (Parameter estimates (*β*) with their standard errors)

Variables	Estimate (*β*)	se	*P*
Intercept	−0·00159	0·00668	<0·001
TCa	0·596	0·0293	<0·001
PP	−0·253	0·0574	<0·05
NPP	0·106	0·0524	<0·001
ExPhyt	0·00172	0·000305	<0·001
TCa×ExPhyt	−0·00693	0·00101	<0·001
TCa×PP	–	–	NS
TCa×NPP	–	–	NS
PP×ExPhyt	0·0121	0·00312	<0·001
PP×NPP	–	–	NS
NPP×ExPhyt	–	–	NS
Pig genotype	–	–	NS
Pig sex	–	–	NS

TCa, total Ca intake; PP, phytate-P intake; NPP, non-phytate-P intake; ExPhyt, exogenous phytase intake.

**Fig. 2:**
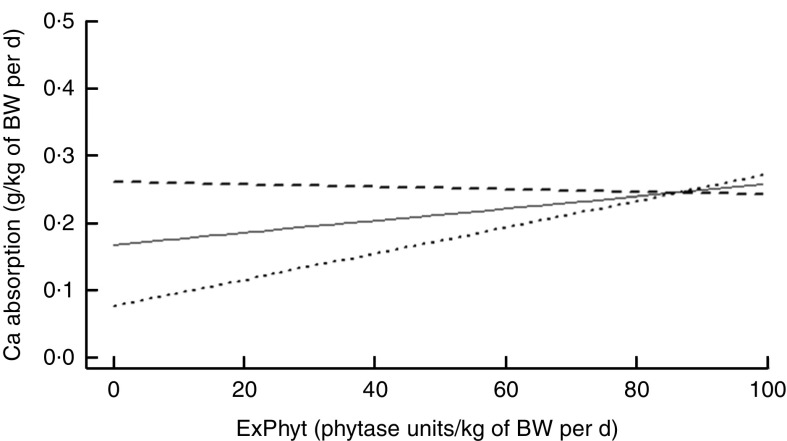
Change in calcium absorption with increasing exogenous phytase intake (ExPhyt) at three different levels of total calcium intake (TCa), to illustrate the interaction between TCa and ExPhyt identified in the final linear mixed effects regression model for calcium absorption. At higher TCa (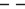: TCa set to its mean+sd from the data set), calcium absorption remains relatively unchanged with increasing ExPhyt. At lower TCa (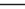: TCa set to its mean value from the data set; 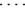: TCa set to its mean−sd from the data set), calcium absorption increases with increasing ExPhyt. The remaining variables (non-phytate-P intake and phytate-P intake) were fixed and set to their mean values from the data set. BW, body weight.

On the basis of the regression, there was an interaction between the effects of TCa and ExPhyt on Ca absorption (*P*<0·001) (Fig. 2), showing that increasing TCa above its mean (0·313 g/kg of BW) reduced Ca absorption at ExPhyt levels exceeding 80 FTU/kg of BW per d. Concurrently, TCa below the mean resulted in an increase in Ca absorption, as ExPhyt increased from 0 to 100 FTU/kg of BW per d.

**Fig. 3:**
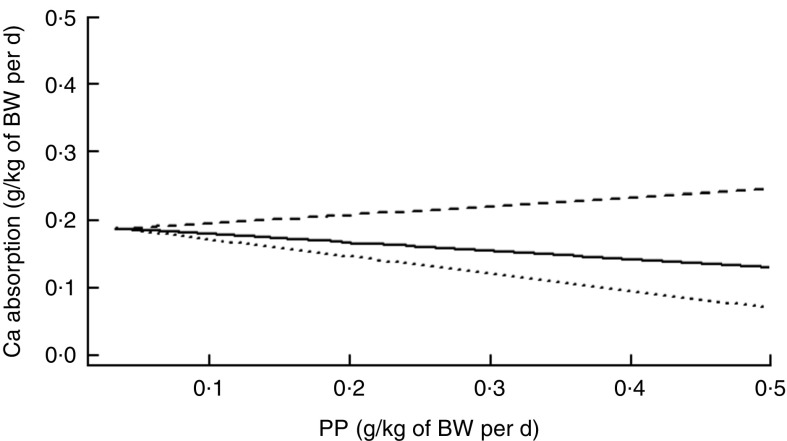
Change in calcium absorption with increasing phytate-P intake (PP) at three different levels of exogenous phytase intake (ExPhyt), to illustrate the interaction between PP and ExPhyt identified in the final linear mixed effects regression model for calcium absorption. At higher ExPhyt (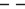: ExPhyt set to its mean+sd from the data set), calcium absorption increases with increasing PP. Lower ExPhyt (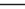: ExPhyt set to its mean value from the data set) and no additional ExPhyt (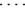) lead to an overall decrease in calcium absorption with increasing PP. The remaining variables (total calcium intake and non-phytate-P intake) were fixed and set to their mean values from the data set. BW, body weight.

There was a further significant interaction between ExPhyt and PP on Ca absorption (*P*<0·001). Although there was a general decrease in Ca absorption with increasing PP at ExPhyt at or below the mean, ExPhyt supplied above the average level helped in facilitating Ca absorption (Fig. 3). There were no other significant interactions in the final LMER model.

**Fig. 4:**
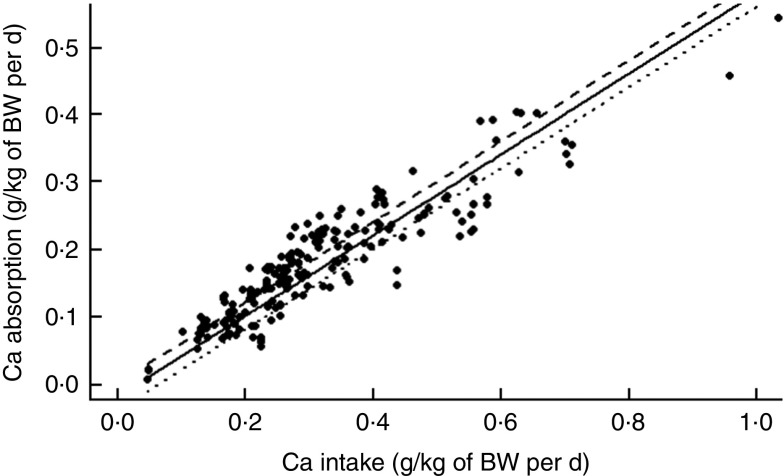
Predicted effects of different phytate-P intake (PP) concentrations expressed as a percentage of total P intake (TP) on calcium absorption for diets containing no additional exogenous phytase intake. 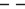, 25 % of TP derived from PP (low PP); 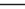, 50 % of TP (medium PP); 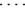, 75 % of TP derived from PP (high PP). TP was set to its mean value from the data set; it is assumed that TP=PP+non-phytate-P intake (NPP), and hence the remaining TP originates from NPP. BW, body weight.

The main fixed effects of TCa, PP, NPP and ExPhyt were all significant (*P*<0·001, *P*<0·05, *P*<0·001 and *P*<0·001, respectively) in the presence of the aforementioned interactions between TCa and ExPhyt, and between ExPhyt and PP. To illustrate how the two types of TP affected Ca absorption, we considered a scenario without any additional ExPhyt. Simplifying the model in this way allows the investigation of separate effects of PP and NPP. In this case, the overall effect of TP on Ca absorption was a net result of the negative effect of PP and the positive effect of NPP. For any given TP, increasing the corresponding PP concentration as a proportion of fixed TP resulted in a decrease in Ca absorption (or vice versa) (Fig. 4).

There were no significant effects of either pig sex or genotype on Ca absorption in the final LMER model. Overall, fixed effect components of the final LMER model explained 90 % of total variability in Ca absorption (*R* ^2^=0·90).

To fully interpret the main fixed effects in the final LMER model with interactions, the role of these fixed effects was examined in a model, where these interactions were excluded. This LMER analysis suggested that Ca absorption increased linearly with increasing TCa (*P*<0·001). TP affected Ca absorption in two contrasting ways, depending on whether it was bound to the phytate molecule. Increasing PP resulted in a marginally significant reduction in Ca absorption (*P*<0·05), whereas increasing NPP had a positive effect on Ca absorption (*P*<0·001). ExPhyt enhanced Ca absorption (*P*<0·001). As in the previous model, there were no effects of animal-related characteristics on the dependent variable.

Similar findings were inferred from weighted linear regression models with cluster robust variances (online Supplementary Material E), demonstrating that the results were unaffected by this change in the method of analysis.

### Analysis of factors affecting calcium retention

The mean Ca retention was 0·158 (se 0·080) g/kg of BW per d, ranging from 0·006 to 0·533 g/kg of BW per d (Table 2). The summary of the significant two-way interactions between the covariates and of the main fixed effects in the final LMER model for Ca retention is presented in Table 5. The interactions identified in the LMER model for Ca absorption were also significant in the model for Ca retention. First, there was a negative interaction between TCa and ExPhyt on Ca retention, suggesting that excessive TCa reduces the efficacy of ExPhyt in aiding Ca retention (*P*<0·001). Second, there was also a positive interaction between the effects of ExPhyt and PP on Ca retention (*P*<0·05).

**Table 5:** Main significant fixed effects and their two-way interactions in the final linear mixed effects regression model for calcium retention (Parameter estimates (*β*) with their standard errors)

Variables	Estimate (*β*)	se	*P*
Intercept	−0·0179	0·00954	<0·001
TCa	0·430	0·0298	<0·001
PP	−0·0188	0·0815	<0·01
NPP	0·313	0·0524	<0·001
ExPhyt	0·00157	0·000298	<0·001
TCa×ExPhyt	−0·00431	0·000870	<0·001
TCa x PP	–	–	NS
TCa×NPP	–	–	NS
PP×ExPhyt	0·00553	0·00258	<0·05
PP×NPP	–	–	NS
NPP×ExPhyt	–	–	NS
Pig genotype	–	–	NS
Pig sex	–	–	NS

TCa, total Ca intake; total P intake; PP, phytate-P intake; NPP, non-phytate-P intake; ExPhyt, exogenous phytase intake.

**Fig. 5:**
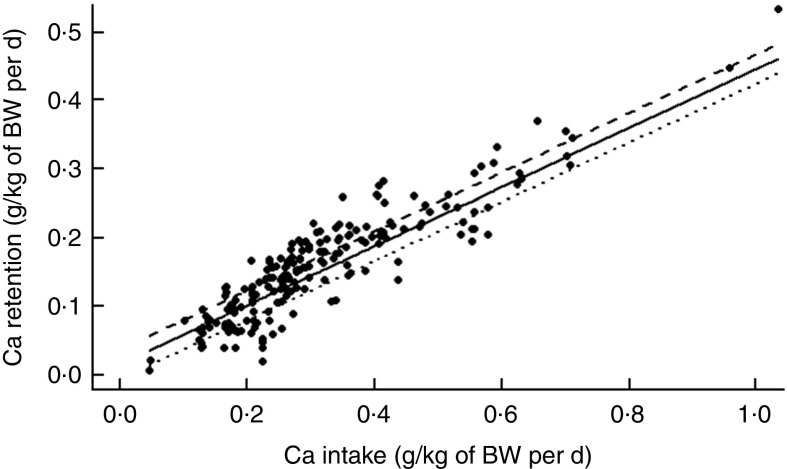
Predicted effects of increasing non-phytate-P intake (NPP) on calcium retention for diets containing no additional exogenous phytase intake. 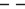, NPP set to its mean+sd from the data set; 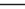, mean NPP from the data set; 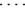, NPP equivalent to its mean−sd from the data set. Phytate-P intake was set to its mean value from the data set for the purposes of this illustration. BW, body weight.

The main fixed effects of TCa, PP, NPP and ExPhyt were statistically significant in the LMER model with interactions (*P*<0·001, *P*<0·01, *P*<0·001 and *P*<0·001, respectively). Ca retention was not sex and breed dependent. To illustrate the predicted, separate effect of NPP on Ca retention, we considered a case without any additional ExPhyt, which simplifies the model. In this instance, increasing NPP led to an overall improvement in Ca retention across the whole TCa range (Fig. 5).

The fixed effects components of the final LMER model with interactions explained 91 % of total variability in Ca retention (*R* ^2^=0·91).

To interpret the main fixed effects in the final LMER model with interactions, the role of these fixed effects was examined in a model in which these interactions were left out. The same factors were statistically significant and qualitatively the same (positive or negative), as for the final LMER for Ca retention with interactions.

Sensitivity analysis based on weighted linear regression models with cluster robust variances (online Supplementary Material E) produced similar outputs, indicating that the above results were unchanged under this different method of analysis.

### Estimation of endogenous calcium losses and obligatory calcium losses

The population average endogenous Ca excretion in pigs fed a Ca-free and P-free diet containing no exogenous phytase was 20·5 mg/kg of BW per d (95 % CI 5·46, 36·5 mg/kg of BW per d; *P*<0·001), whereas the estimated obligatory Ca losses were 28·6 mg/kg of BW per d (95 % CI 7·79, 49·5; *P*<0·001). When expressed on a DMI basis, the average endogenous Ca excretion was 239 mg/kg of DMI (95 % CI 114, 364 mg/kg of BW per d; *P*<0·001), and the average obligatory Ca losses were 286 mg/kg of DMI (95 % CI 124, 449 mg/kg of DMI; *P*<0·001). A summary of endogenous Ca losses (in mg/kg of BW per d) reported in the literature, evaluated for dietary treatments with a variable dietary P content, along with the meta-analytic estimates of endogenous and obligatory Ca losses incorporating this information is shown in Table 6. A summary of estimated endogenous Ca losses expressed on a DMI basis is presented in Table 7.

**Table 6:** Summary of endogenous calcium losses (mg/kg of body weight (BW) per d) reported in the literature along with the estimated study-specific endogenous calcium excretion and obligatory calcium losses based on the meta-regression*

References	Method	Dietary intake†			Reported endogenous	Estimated	Estimated
First author	Type of estimation	TCa (g/kg of BW per d)	TP (g/kg of BW per d)	ExPhyt (phytase units/kg of BW per d)	Ca losses (mg/kg of BW per d)‡	endogenous Ca losses (mg/kg of BW per d)§	obligatory Ca losses (mg/kg of BW per d)||
González-Vega *et al*.^(^ ^14^ ^)^	Linear regression	–	0·258	–	7·18	9·47	21·8
González-Vega *et al*.^(^ ^15^ ^)^	Semi-synthetic Ca-free diet based on maize starch, potato protein isolate, soyabean oil and sucrose	0·00844	0·152	–	8·46	15·4	21·52
Merriman & Stein^(^ ^16^ ^)^	Semi-synthetic Ca-free diet based on maize starch, potato protein isolate, soyabean oil and sucrose	0·00784	0·172	–	12·8	15·6	21·2
González-Vega *et al*.^(^ ^15^ ^)^	Semi-synthetic Ca-free diet based on maize starch, potato protein isolate, soyabean oil and sucrose	0·0142	0·227	–	17·1	15·1	17·43

TCa, total Ca intake; TP, total P intake; ExPhyt, exogenous phytase intake; LMER, linear mixed effects regression.

* References are presented from smallest to largest reported values.

† Calculated by dividing the reported intakes of Ca and P (g/d) and exogenous phytase (phytase units/d) by the reported initial BW (kg) at the start of each experiment.

‡ Calculated by dividing the reported endogenous loss (mg/d) by the reported initial BW (kg).

§ Calculated based on the following population level LMER equation: Ca absorption=−0·0205+0·527TCa+0·0428TP+0·00192ExPhyt−0·0112TCa×ExPhyt + 0·0105TP×ExPhyt; all variables are expressed on g/kg of BW per d basis.

|| Calculated based on the following population level LMER equation: Ca retention=−0·0286+0·365TCa+0·0266TP+0·00202ExPhyt−0·00779TCa×ExPhyt + 0·00493TP×ExPhyt; all variables are expressed on g/kg of BW per d basis.

**Table 7:** Summary of endogenous calcium losses (mg/kg of DM intake (DMI)) reported in the literature along with the estimated endogenous calcium excretion and obligatory calcium losses based on the meta-regression*

References	Method		Estimated endogenous Ca losses	Estimated obligatory Ca losses
First author	Type of estimation	Initial BW range (kg)	(mg/kg of DMI)†	(mg/kg of DMI)‡
González-Vega *et al*.^(^ ^14^ ^)^	Linear regression	14·2–19·2	160	–
González-Vega *et al*.^(^ ^15^ ^)^	Semi-synthetic Ca-free diet based on maize starch, potato protein isolate, soyabean oil and sucrose	17·1–21·2	220	–
This study	Meta-regression	19·9–75·0	239†	287‡
Merriman & Stein^(^ ^16^ ^)^	Semi-synthetic Ca-free diet based on maize starch, potato protein isolate, soyabean oil and sucrose	14·7–16·1	329	–
González-Vega *et al*.^(^ ^15^ ^)^	Semi-synthetic Ca-free diet based on maize starch, potato protein isolate, soyabean oil and sucrose	18·4–20·4	396	–

BW, body weight; LMER, linear mixed effects regression; TCa, total Ca intake; TP, total P intake; ExPhyt, exogenous phytase intake.

* References are presented in order of the estimated magnitude.

† Calculated based on the following population level LMER equation: Ca absorption=−0·239+0·541TCa+0·0171TP+0·00151ExPhyt−0·0000483TCa×ExPhyt + 0·000113TP×ExPhyt; all variables are expressed on g/kg of DMI basis.

‡ Calculated based on the following population level LMER equation: Ca retention=−0·287+0·455TCa+0·111TP+0·000440ExPhyt−0·000157TCa×ExPhyt + 0·000220TP×ExPhyt; all variables are expressed on g/kg of DMI basis.

### Estimation of requirement for maintenance and gross efficiency of utilisation

On the basis of the results of the LMER model for Ca retention with TCa, TP and ExPhyt as covariates and a random effect associated with each study, the average total Ca requirement for maintenance in the context of Ca- and P-free diet was estimated as 78·5 mg/kg of BW per d and ranged from 21·4 to 135 mg/kg of BW per d. The estimated gross efficiency of Ca utilisation was 36·5 % (95 % CI 30·8, 42·2 %; *P*<0·001).

## Discussion

Over the past 10 years, the industry has been formulating diets based on the ratio of total Ca:digestible P to limit the environmental impact associated with excessive P excretion, while attempting to ensure that pig performance and health are maintained. However, formulating feed diets based on total Ca has major disadvantages, because, as the exact Ca requirements are unknown, diets may not meet Ca requirements and have a negative impact on animal performance, as well as feed conversion^(^ ^51^ ^)^. Developing recommendations based on the digestible, as opposed to the total Ca values, is an essential step to optimise both Ca and P utilisation, further minimise P excretion, as well as to improve growth and bone health in pigs^(^ ^13^ ^)^. A lack of information on the subject of Ca digestibility and utilisation in growing and finishing pigs is reflected in the existing scientific literature; although the number of reported Ca balance studies does not exceed double figures, its P equivalent consists of several hundred studies. Reviews focusing on P digestion and metabolism have been previously written^(^ ^52^ ^,^ ^53^ ^)^, but to our knowledge there is no comparable analysis for Ca. Recent experiments have been able to determine digestible Ca values for several organic and inorganic sources^(^ ^14^ ^–^ ^17^ ^)^, but more work is needed to further advance the current understanding of Ca digestibility and utilisation. Therefore, a systematic review and meta-analysis were carried out to address this knowledge gap and provide an expansion to the existing body of literature. A meta-analytic approach was chosen to synthesise the data, as it provides a more formal and robust way of quantifying previously published results than qualitative summaries of literature^(^ ^54^ ^)^.

This study identified the direct and interactive effects of *a priori* factors affecting Ca digestibility and utilisation. Specifically, the results of the current meta-analysis confirmed that Ca absorption and retention are complex processes dependent upon Ca, PP, NPP, exogenous phytase and some interactions between these factors. The high level of variance in Ca absorption (90 %) and Ca retention (91 %) explained by the models supported the choice of independent variables selected at the outset of this meta-analysis and confirmed that these processes are predominantly affected by the above-mentioned dietary characteristics.

Although it is widely accepted that exogenous phytase improves P absorption in pigs^(^ ^55^ ^)^ by increasing bioavailability of PP^(^ ^56^ ^)^, the exact effects of this supplementation on Ca digestibility and utilisation are not clear. Several authors suggested that exogenous phytase could help with Ca digestibility^(^ ^57^ ^,^ ^58^ ^)^, whereas others found no evidence supporting this claim ^(^ ^59^ ^,^ ^60^ ^)^. The current findings indicate that exogenous phytase is not only consequential for P metabolism but also affects Ca metabolism through improvements in Ca absorption and retention. For example, based on the typical Ca and TP (with a proportion of PP set to 60 %) meeting the current National Research Council^(^ ^19^ ^)^ guidelines for a 25-kg pig, an exogenous phytase supplementation of 1000 FTU per d may improve Ca digestibility by approximately 20–25 %. The positive interaction between exogenous phytase and PP identified in the Ca absorption and retention models can be linked to a reduction in the formation of insoluble, indigestible Ca–PP complexes^(^ ^20^ ^)^, as the PP molecule was previously shown to chelate with Ca^(^ ^61^ ^)^. On the basis of the current data, increasing PP intake in diets containing either suboptimal or no exogenous phytase supplementation had detrimental effects on Ca absorption and retention. As an illustration, increasing the proportion of PP from 50 to 75 % of the TP in such diets may lead to a 10–15 % reduction in Ca digestibility. However, the results of the present study show that in the presence of exogenous phytase this negative impact of PP could be potentially neutralised.

On the other hand, previous studies^(^ ^62^ ^,^ ^63^ ^)^ indicated that exogenous phytase is most effective in increasing P absorption at lower levels of dietary Ca. The current results demonstrate that this may also hold true with respect to Ca digestibility. At higher Ca intakes, the response of Ca absorption and retention was almost insensitive to increased levels of exogenous phytase supplementation. In contrast, at lower Ca intakes, the positive effect of increasing exogenous phytase was much more prominent and resulted in improved Ca utilisation through an increase in the provision of digestible Ca. Notably, at exogenous phytase doses exceeding 1000 FTU per d, a reduction in Ca consumption from 120 to 80 % of current National Research Council^(^ ^19^ ^)^ guidelines for total Ca yielded comparable absorption and retention values and hence led to a decrease in Ca excretion. These findings provide further evidence that excess Ca reduces the efficacy of exogenous phytase and are consistent with the results by Lei *et al*.^(^ ^64^ ^)^ in weanling pigs. This negative interaction between increasing Ca intake and exogenous phytase can certainly be linked to the aforementioned Ca–PP complexes, which are more likely to form in the presence of abundant Ca. Other plausible mechanisms explaining this interaction between Ca and exogenous phytase identified in the current meta-analysis relate to either potential changes in the gastrointestinal pH^(^ ^58^ ^)^ or Ca directly repressing activity of this supplement by competing for active sites of the enzyme^(^ ^65^ ^)^.

On the basis of the present results, it could be concluded that the addition of exogenous phytase may contribute towards a reduction of excess inorganic Ca supplementation, which in turn may increase P digestibility^(^ ^66^ ^)^. These results also indicate that reducing dietary Ca in the presence of this enzyme may promote more efficient use of Ca by the animal. Hence, a more accurate dietary formulation should be achieved if Ca requirements were expressed on a digestible Ca basis. Moreover, as Ca digestibility was shown to increase with exogenous phytase supplementation, this factor should also be taken into account when formulating diets. Nevertheless, caution should be exercised, as in certain scenarios an increase in Ca digestibility could widen the Ca:P ratio to an extent, where it negatively affects growth and bone mineralisation^(^ ^67^ ^,^ ^68^ ^)^. Incidentally, Létourneau-Montminy *et al*.^(^ ^69^ ^)^ also advocated reducing dietary Ca levels to optimise growth performance in weaned piglets, but a deficit in dietary Ca could have an adverse effect on bone health^(^ ^70^ ^,^ ^71^ ^)^. Owing to an insufficient number of publications reporting performance data together with Ca balance data, it was impossible to draw inferences about the effects of varying Ca intake on animal growth and bone health in this meta-analysis.

Animal characteristics did not seem to affect Ca digestibility and utilisation. In particular, Ca absorption and retention were not breed dependent. This perceived absence of pig genotype effect could reflect the over-representation of LW cross-breeds, and the consequent under-representation of other breeds in the present data set. Similar findings were reported by Douglas *et al*.^(^ ^40^ ^)^, albeit in relation to animal performance and feed efficiency, rather than mineral digestibility.

In addition to the analysis of factors affecting Ca absorption and retention, estimates of total Ca requirement for maintenance, gross efficiency of Ca utilisation, endogenous Ca excretion and obligatory Ca losses were derived. To our knowledge, this is the first attempt at evaluating these quantities in growing and finishing pigs using a meta-analytic approach. On the basis of the data from previous digestibility trials, the average total Ca requirement for maintenance was 78·5 mg/kg of BW per d and ranged from 21·4 to 136 mg/kg of BW per d. Evidently, this estimate has a wide range and may be partly explained by the choice of the scaling unit (BW), which poses limitations. As body mass contains protein, water, lipid and ash^(^ ^72^ ^)^, this scaling factor includes body fat, which can greatly vary between different pigs. As lipid reserves do not require any Ca, it is expected that the maintenance requirement calculated in this manner will be over-estimated for pigs with a higher body fat. As the data set was built from multiple experiments carried out on over 1000 pigs over a period of 22 years, it is prudent to assume that there is a large variability in the levels of fatness among these animals, which is then reflected in a large CI associated with our estimate. Consequently, Emmans & Kyriazakis^(^ ^73^ ^)^ outlined potential advantages of expressing the maintenance requirement in terms of body protein mass, rather than body weight, to account for this matter. The body protein mass is difficult to measure *in vivo*, and it was not reported in any of the selected studies. Hence, it was not feasible to use this alternative scalar in the current study.

The information on the subject of endogenous losses of Ca is sparse and limited to only a handful of publications, which hampers the development of precise recommendations to satisfy Ca requirements based on a digestible Ca basis. Furthermore, these reported estimates are highly variable, and seem to be strongly dependent on the choice of methodology, as well as dietary ingredients used in formulating experimental treatments. This variability is omnipresent, even when considerably higher mineral excretion estimates measured via radioactive isotope dilution reported in older studies^(^ ^74^ ^–^ ^76^ ^)^ are excluded, and only results obtained by the currently preferred methods (linear regression, or measuring faecal Ca outputs in pigs fed semi-synthetic Ca-free diets) are examined. In the present study, two estimates of the endogenous Ca excretion were derived: one scaled by DMI and the other related to body weight. The former approach enables derivation of endogenous Ca losses, which literature sources^(^ ^19^ ^,^ ^58^ ^)^ state are expected to be independent of dietary characteristics, and which can be directly incorporated in diet formulations. The latter method yields a dynamic estimate of the mineral excretion, which can be readily adjusted according to the daily weight gain of an individual pig, but is diet-dependent. On the basis of the data, the average endogenous excretion was estimated as 239 mg/kg of DMI (95 % CI 114, 364 mg/kg of BW per d), which is comparable with the range of values previously reported in the literature, from 160^(^ ^14^ ^)^ to 396^(^ ^15^ ^)^ mg/kg of DMI. In addition, when scaled by body weight, the average endogenous Ca losses in Ca- and P-free diets containing no additional phytase were 20·5 mg/kg of BW per d. However, care should be taken when comparing this particular estimate with values reported in the literature^(^ ^14^ ^–^ ^16^ ^)^, as these experiments did not evaluate endogenous losses for diets simultaneously free from both Ca and P. Consequently, when dietary P intake information was incorporated to estimate the study-specific endogenous Ca excretion reflecting each experimental dietary treatment, meta-analytic estimates ranged from 9·47 to 15·6 mg/kg of BW per d and were lower than the predicted losses in a Ca- and P-free diet. These results imply that dietary P may be an important source contributing to the variability in endogenous Ca losses reported in the literature.

The difference between the estimated obligatory and endogenous losses (amounting to 48·0 mg/kg of DMI) indicates that there are considerable urine losses, which could be incorporated to devise an additional set of Ca requirements based on retained Ca. Hence, an alternative system of dietary formulation in growing and finishing pigs could be developed by combining obligatory Ca losses derived in this study and equivalent P losses reported by Schulin-Zeuthen *et al*.^(^ ^52^ ^)^.

The average gross efficiency of total Ca utilisation was calculated to equal 36·5 % and is comparable to the gross efficiency of total P utilisation obtained by other researchers^(^ ^52^ ^,^ ^77^ ^,^ ^78^ ^)^. However, a lack of information concerning the Ca digestibility (and hence utilisation) of individual feed ingredients and the complete diets makes it difficult to fully interpret our estimate. For example, González-Vega *et al*.^(^ ^79^ ^)^ determined an apparent digestibility in Ca carbonate to be 58 %, which is significantly higher than the 38 % obtained by Kemme *et al*.^(^ ^18^ ^)^ in the context of limestone. Other than the Ca source itself, one plausible explanation for this large variability between these estimates may be owing to the influence of PP in the diets and may also explain a relatively low gross efficiency of Ca utilisation derived in this study. Moreover, the influence of PP will be further amplified if diets are formulated to contain excess dietary Ca levels. This issue is yet another indication that the current system of dietary recommendations based on total Ca values may have a significant negative impact on feed efficiency.

## Conclusions

This meta-analysis sought to clarify and update the current knowledge on Ca digestibility and utilisation in growing and finishing pigs. The outcomes of this study may help establish requirements for digestible Ca. Formulating diets based on digestible Ca, instead of total Ca values, will probably benefit the pig industry by optimising both Ca and P utilisation, as well as limiting the potential environmental damage associated with the excess P excretion.

Interactions between Ca, P and exogenous phytase influenced Ca absorption and retention. The inclusion of exogenous phytase seemed to improve Ca digestibility and hence may lead to an overall reduction of excess inorganic Ca supplementation. In the presence of this enzyme, the negative effect of PP on Ca absorption and retention may be neutralised. However, it was demonstrated that excess Ca may reduce the efficacy of exogenous phytase. Overall, these results highlight that the effects of exogenous phytase should be taken into account in any future diet formulations based on a digestible Ca basis.

The aforementioned nutrient interactions may also affect estimation of endogenous Ca losses, which may explain large variability in values previously reported in the literature. Our models are able to account for these potential sources of variability by incorporating these factors during the estimation process. Furthermore, the present estimate of endogenous Ca losses expressed in relation to DMI may be used to calculate standardised total tract digestibility of Ca, which in turn would enable Ca to be treated in the same manner as P in diet formulations.

However, this meta-analysis was restricted by the amount of information on the digestibility and utilisation of Ca; this justifies the view that Ca is usually neglected when it comes to feed formulation for growing pigs. The outcomes of this paper may provide the impetus for estimating these quantities, and thus accounting for requirements, as well as the factors that affect this important nutrient.

Our study highlighted the complexity associated with the subject of Ca digestibility and its dependence upon several dietary characteristics. Other methodologies, such as mechanistic modelling, may be used to further develop an understanding of the aforementioned interactions and their effect on the utilisation of both Ca and P.

## Supplementary material

For supplementary material accompanying this paper visit http://dx.doi.org/10.1017/S0007114518000612. click here to view supplementary material
