# Supplementary material for: Do not neglect calcium: a systematic review and meta-analysis (meta-regression) of its digestibility and utilisation in growing and finishing pigs
Source: Br J Nutr. 2018 Apr 3;119(11):1207–19. doi: 10.1017/S0007114518000612 (PMC5960347; doi:10.1017/S0007114518000612)
Supplement: Supplementary file 1 [file S0007114518000612sup001.docx]

**Supplementary Material A: Research protocol for the systematic review and meta- analysis of Ca digestibility and utilisation in growing and finishing pigs**

1. **Review question(s):**

**Primary questions**

- How are Ca digestibility and utilisation affected by the dietary characteristics, such as phytate-P, non-phytate-P, exogenous phytase and Vitamin D?
- How are Ca digestibility and utilisation affected by the animal related characteristics, such as genotype and gender?

**Secondary questions**

- What is the response of Ca digestibility and utilisation to varying dietary Ca intake levels?
- What are the Ca requirements for maintenance in growing and finishing pigs? Is it possible to derive estimates for endogenous loss of Ca and its efficiency of utilisation?
- What is the effect of increasing Ca levels on pig performance?

1. **Searches:**

**Peer reviewed journals:** following electronic databases will be searched for peer-reviewed journals: The Web of Science and Scopus

**Grey literature:** 1) Google Scholar and Science.gov will be utilised to find any relevant data; first 250 hits for each search engine will be considered. 2) Materials issued by public bodies (e.g. EFSA, USDA) will be reviewed. 3) Key lead authors will be contacted via emails

1. **Keyword searches for the Web of Science/Scopus databases:**

- Calcium AND digest* OR utilisation OR utilization OR absor* OR metabol* OR require* OR level* OR concentration* AND (pig NOT guinea pig) OR swine
- Calcium AND ratio OR percent*OR rate OR proportion AND (pig NOT guinea pig) OR swine
- Calcium AND Phosp* AND digest* OR utilisation OR utilization OR absor* OR metabol* OR require* OR level* OR concentration* AND (pig NOT guinea pig) OR swine
- Calcium AND Phosp* AND ratio OR percent*OR rate OR proportion AND (pig NOT guinea pig) OR swine
- Calcium AND Vitamin D AND digest* OR utilisation OR utilization OR absor* OR metabol* OR require* OR level* OR concentration* AND (pig NOT guinea pig) OR swine
- Calcium AND Vitamin D AND ratio OR percent*OR rate OR proportion AND (pig NOT guinea pig) OR swine
- Calcium AND Phytase AND digest* OR utilisation OR utilization OR absor* OR metabol* OR require* OR level* OR concentration* AND (pig NOT guinea pig) OR swine
- Calcium AND Phytase AND ratio OR percent*OR rate OR proportion AND (pig NOT guinea pig) OR swine

Timespan of searches: 1990 – present day

1. **Types of study to be included:** any in-vivo experimental study design presenting relevant data for Ca and P intakes, Ca and P outputs from faeces and urine, Ca and P absorption/retention values on relevant population outlined in part 7 of this research protocol (if a paper reports mineral intake values and mineral outputs from faeces and urine, mineral absorption and retention will be calculated by the reviewer, if necessary)
2. **Condition/domain being studied:** growing and finishing pigs fed different dietary levels of Ca and P (with and without exogenous phytase supplementation); growing and finishing pigs fed different dietary Ca: P ratios (weanling pigs will be also considered if there is insufficient data available for growing and finishing pigs)
3. **Participants/population:**

**Inclusion:** growing (pigs that overcame stress associated with weaning) and finishing pigs (Initial BW range: 50-100kg)

**Exclusion:** breeding sows, gestating sows, farrowing sows, new-born piglets, weanling piglets, weaned piglets that have not overcame stress associated with weaning

| **Component** | **Inclusion** | **Exclusion** |
| --- | --- | --- |
| **Study design:** | quantitative, in-vivo: mineral balance studies | descriptive, qualitative, in-silico, in-vitro |
| **Population:** | growing and finishing pigs | new-born piglets, weanling piglets |
| **Subpopulation:** | barrow (castrated male), boar (entire male), gilt | breeding sow, gestating sow, farrowing sow |
| **Treatment:** | varying levels of dietary Ca and P (with and without exogenous phytase supplementation | - |
| **Primary outcomes:** | Ca absorption and Ca retention | other available measures of mineral digestibility |

1. **Intervention(s), exposure(s):** different dietary Ca levels in the diets of growing and finishing pigs (for different dietary P levels) with and without exogenous phytase supplementation
2. **Outcomes:**

**Primary outcomes:** Ca absorption and Ca retention (mean, confidence intervals, standard errors) at varying dietary Ca intakes

**Secondary outcomes:** Growth performance (Average Daily Gain, bone mineralisation, bone breaking strength) at varying dietary Ca intakes (mean, confidence intervals, standard errors)

1. **Data extraction:** First keyword searches will be performed and results of these searches will be exported into unfiltered reference library; duplicates will be removed. Next title and abstracts will be examined according to the inclusion/exclusion criteria listed in part 6 of this research protocol. Afterwards kappa assessments will be calculated and possible relevant papers will be moved to a separate, filtered reference library. These articles will be examined in full to determine whether or not they can be selected for further data analysis. References of papers included for further data analysis will also be scanned to find any relevant studies. The following data will be extracted into a purpose – built database: 1) First author, 2) year of publication, 3) location of publication, 4) dietary Ca and P concentrations, 5) dietary Vitamin D, 6) dietary exogenous phytase, 7) Ca and P intakes, 8) Ca outputs from faeces and urine, 9) Ca absorption and retention, 10) exogenous phytase type (if used), 11) main source of dietary protein, 12) main source of energy, 13) pig breed, 14) pig sex, 15) sample sizes per treatment, 16) measure of variability (e.g. standard error associated with main Ca absorption and retention), 17) diet type, 18) growth performance (average daily gain, bone mineralisation, bone breaking strength), 19) other factors considered in the study
2. **Risk of bias (quality) assessment**: A **s**econd reviewer will be invited to go through a subsample of articles from the initial list produced by the relevant literature search and kappa assessment will be performed. A kappa rating of 0.6 or higher is recommended to pass the assessment. Afterwards, the selected studies will be assessed on the following criteria: 1) selection bias – is there any allocation concealment; 2) attrition bias – how did the study deal with the incomplete data and dropouts (e.g. pigs that have not survived the full length of the experiment); 3) reporting bias – are there any occurrences of any selective outcome reporting? Special care must be taken who the studies examine the effects of exogenous phytase supplementation for possible conflict of interest. Critical appraisal of the data will be then performed to determine the overall quality of the published data. Data reported in the included studies will be checked for omissions and internal consistency.
3. **Strategy for data synthesis and analysis:** 1) testing for heterogeneity will be performed to ensure that the assumption that all studies come from the same population is reasonable; if this assumption is rejected, due to the nature of the data considered for the purposes of this systematic review (input-output), mixed effect regression will be used to analyse the data; 2) data will be weighed by the measure of variability reported per treatment

**Supplementary Material B: Detailed results of the critical appraisal**

1. Risk of bias for selected studies, assessed using the SYRCLE’s risk of bias tool

| **Study** | **A. Selection bias: sequence generation** | **B. Selection bias: baseline characteristics** | **C. Selection bias: allocation concealment** | **D. Performance bias: random housing** | **E. Performance bias: blinding** | **F. Detection bias: random outcome assessment** | **G. Detection bias: blinding** | **H. Attirtion bias: incomplete outcome data** | **I. Reporting bias: selective outcome reporting** | **J. Other sources of bias: methodological bias** |
| --- | --- | --- | --- | --- | --- | --- | --- | --- | --- | --- |
| Jolliff and Mahan^(1)^ | **?** | **✓** | **x** | **?** | **x** | **x** | **x** | **✓** | **✓** | **x** |
| Gutzwiller et al.^(2)^ | **?** | **✓** | **x** | **?** | **x** | **x** | **x** | **✓** | **✓** | **x** |
| Stein et al.^(3)^ | **?** | **✓** | **x** | **?** | **x** | **x** | **x** | **✓** | **✓** | **x** |
| Liao et al.^(4)^ | **✓** | **✓** | **x** | **?** | **x** | **x** | **x** | **✓** | **✓** | **x** |
| Fan et al.^(5)^ | **?** | **✓** | **x** | **?** | **x** | **x** | **x** | **✓** | **✓** | **x** |
| Carter et al.^(6)^ | **?** | **✓** | **x** | **?** | **x** | **x** | **x** | **✓** | **✓** | **x** |
| Li et al.^(7)^ | **✓** | **✓** | **x** | **?** | **x** | **x** | **x** | **✓** | **✓** | **x** |
| Meidinger et al.^(8)^ | **x** | **✓** | **x** | **?** | **x** | **x** | **x** | **✓** | **✓** | **x** |
| Poulsen et al.^(9)^ | **x** | **✓** | **x** | **?** | **x** | **x** | **x** | **✓** | **✓** | **x** |
| Nørgaard et al.^(10)^ | **?** | **✓** | **x** | **?** | **x** | **x** | **x** | **✓** | **✓** | **x** |
| Stein et al.^(11)^ | **?** | **✓** | **x** | **?** | **x** | **x** | **x** | **✓** | **✓** | **x** |
| Pointillart et al.^(12)^ | **?** | **✓** | **x** | **?** | **x** | **x** | **x** | **✓** | **✓** | **x** |
| O’Doherty et al.^(13)^ | **?** | **✓** | **x** | **?** | **x** | **x** | **x** | **✓** | **✓** | **x** |
| Merriman and Stein^(14)^ | **✓** | **✓** | **x** | **?** | **x** | **x** | **x** | **✓** | **✓** | **x** |
| Blaabjerg et al.^(15)^ | **?** | **✓** | **x** | **?** | **x** | **x** | **x** | **✓** | **✓** | **x** |
| Kraler et al.^(16)^ | **?** | **✓** | **x** | **?** | **x** | **x** | **x** | **✓** | **✓** | **x** |
| Létourneau-Montminy et al.^(17)^ | **?** | **✓** | **x** | **?** | **x** | **x** | **x** | **✓** | **✓** | **x** |
| Liu et al.^(18)^ | **?** | **✓** | **x** | **?** | **x** | **x** | **x** | **✓** | **✓** | **x** |
| Blaabjerg et al.^(19)^ | **?** | **✓** | **x** | **?** | **x** | **x** | **x** | **✓** | **✓** | **x** |
| Jolliff and Mahan^(20)^ | **?** | **✓** | **x** | **?** | **x** | **x** | **x** | **✓** | **✓** | **x** |
| Almeida and Stein^(21)^ | **?** | **✓** | **x** | **?** | **x** | **x** | **x** | **✓** | **✓** | **x** |
| Sauer W et al.^(22)^ | **?** | **✓** | **x** | **?** | **x** | **x** | **x** | **✓** | **✓** | **x** |
| Pomar C et al.^(23)^ | **?** | **✓** | **x** | **?** | **x** | **x** | **x** | **✓** | **✓** | **x** |
| Salobir et al.^(24)^ | **?** | **✓** | **x** | **?** | **x** | **x** | **x** | **✓** | **✓** | **x** |
| Sauer et al.^(25)^ | **✓** | **✓** | **x** | **?** | **x** | **x** | **x** | **✓** | **✓** | **x** |
| Veum et al.^(26)^ | **?** | **✓** | **x** | **?** | **x** | **x** | **x** | **✓** | **✓** | **x** |
| Veum et al.^(27)^ | **?** | **✓** | **x** | **?** | **x** | **x** | **x** | **✓** | **✓** | **x** |
| Larsen et al.^(28)^ | **?** | **✓** | **x** | **?** | **x** | **x** | **x** | **✓** | **✓** | **x** |
| Han et al.^(29)^ | **x** | **✓** | **x** | **?** | **x** | **x** | **x** | **✓** | **✓** | **x** |
| Näsi et al.^(30)^ | **✓** | **✓** | **x** | **?** | **x** | **x** | **x** | **✓** | **✓** | **x** |
| Jang et al.^(31)^ | **✓** | **✓** | **x** | **?** | **x** | **x** | **x** | **✓** | **✓** | **x** |
| González-Vega et al.^(32)^ | **✓** | **✓** | **x** | **?** | **x** | **x** | **x** | **✓** | **✓** | **x** |
| Adhikari et al.^(33)^ | **?** | **✓** | **x** | **?** | **x** | **x** | **x** | **✓** | **✓** | **x** |
| Nørgaard et al.^(34)^ | **?** | **✓** | **x** | **?** | **x** | **x** | **x** | **✓** | **✓** | **x** |
| Liao et al.^(35)^ | **?** | **✓** | **x** | **?** | **x** | **x** | **x** | **✓** | **✓** | **x** |
| Korniewicz et al.^(36)^ | **x** | **✓** | **x** | **?** | **x** | **x** | **x** | **✓** | **✓** | **x** |
| Hu et al.^(37)^ | **?** | **✓** | **x** | **?** | **x** | **x** | **x** | **✓** | **✓** | **x** |
| Rideout and Fan^(38)^ | **?** | **✓** | **x** | **?** | **x** | **x** |  | **✓** | **✓** | **x** |
| Nortey et al.^(39)^ | **?** | **✓** | **x** | **?** | **x** | **x** | **x** | **✓** | **✓** | **x** |
| Adeola^(40)^ | **n/a** | **n/a** | **n/a** | **n/a** | **n/a** | **n/a** | **n/a** | **n/a** | **n/a** | **x** |

1. **✓** - adequate randomisation with a clear description of a random component in the sequence generation process/no randomisation necessary due to an experiment design (i.e. Latin square design); **?** - randomised but no details; **x** - no evidence of randomisation; **n/a -** unknown (grey literature: unpublished results)
2. **✓** - baseline characteristics given; **x** -baseline characteristics not given; **n/a -** unknown (grey literature: unpublished results)
3. **✓** - evidence of adequate concealment of groups; **x** - no evidence of adequate concealment of groups; **n/a** **-** unknown (grey literature: unpublished results)
4. **✓** - evidence of random housing of animals; **?** – unclear housing arrangements; **x** - no evidence of random housing of animals; **n/a** **-** unknown (grey literature: unpublished results)
5. **✓** - evidence of caregivers blinded to intervention; **x** - no evidence of caregivers blinded to intervention; **n/a -** unknown (grey literature: unpublished results)
6. **✓** - evidence of random selection for assessment; **x** - no evidence of random selection for assessment; **n/a -** unknown (grey literature: unpublished results)
7. **✓** - evidence of assessor blinded; **x** - no evidence of assessor blinded; **n/a -** unknown (grey literature: unpublished results)
8. **✓** - no missing data/sufficient explanation of missing data; **x** - no explanation of missing data; **n/a** **-** unknown (grey literature: unpublished results)
9. **✓** - free of selective reporting based on methods/results; **x** - selective reporting; **n/a** **-** unknown (grey literature: unpublished results)

| 1. **✓** - free of methodological bias; **x** - evidence of methodological bias |
| --- |

1. Bar chart visualising risk of bias for selected studies, assessed using the SYRCLE’s risk of bias tool

**Supplementary Material C: Examples of diagnostic regression plots**

1. QQ plot of residuals obtained from the fixed effects component of the final LMER model for Ca Absorption


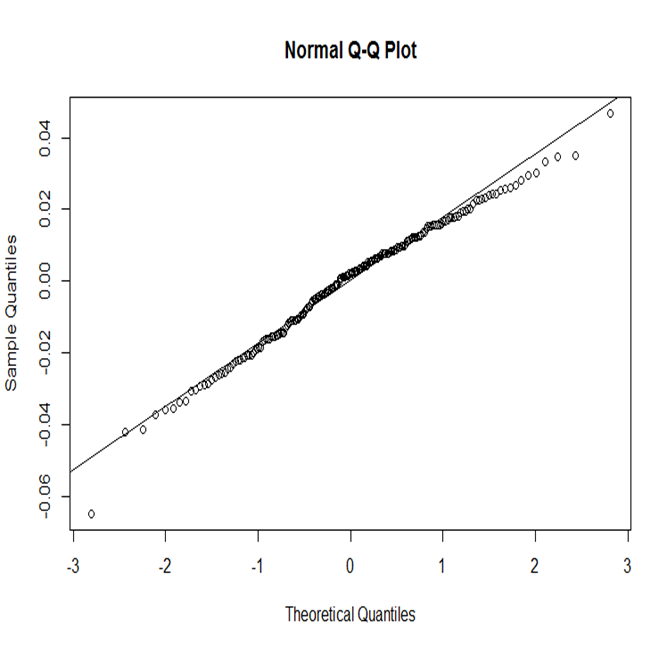


1. Plot of residuals vs fitted values from the fixed effects component of the final LMER model for Ca absorption


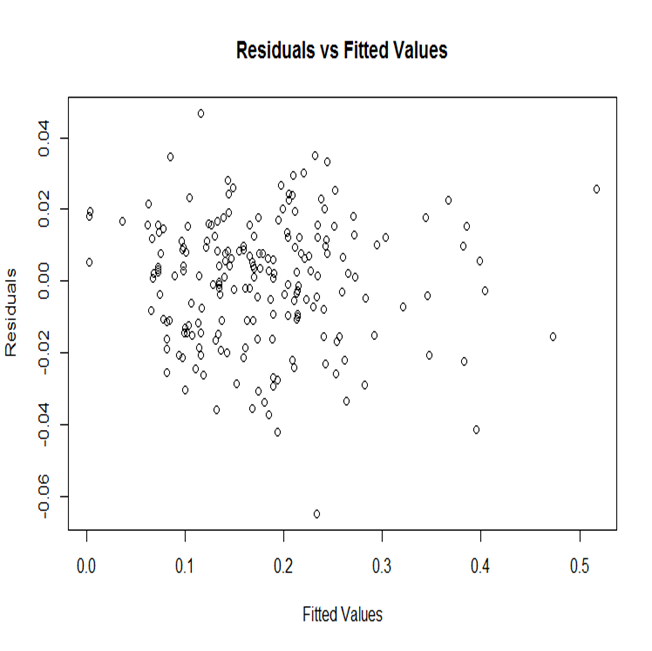


1. Plot assessing normality of random effects of the final LMER model for Ca absorption


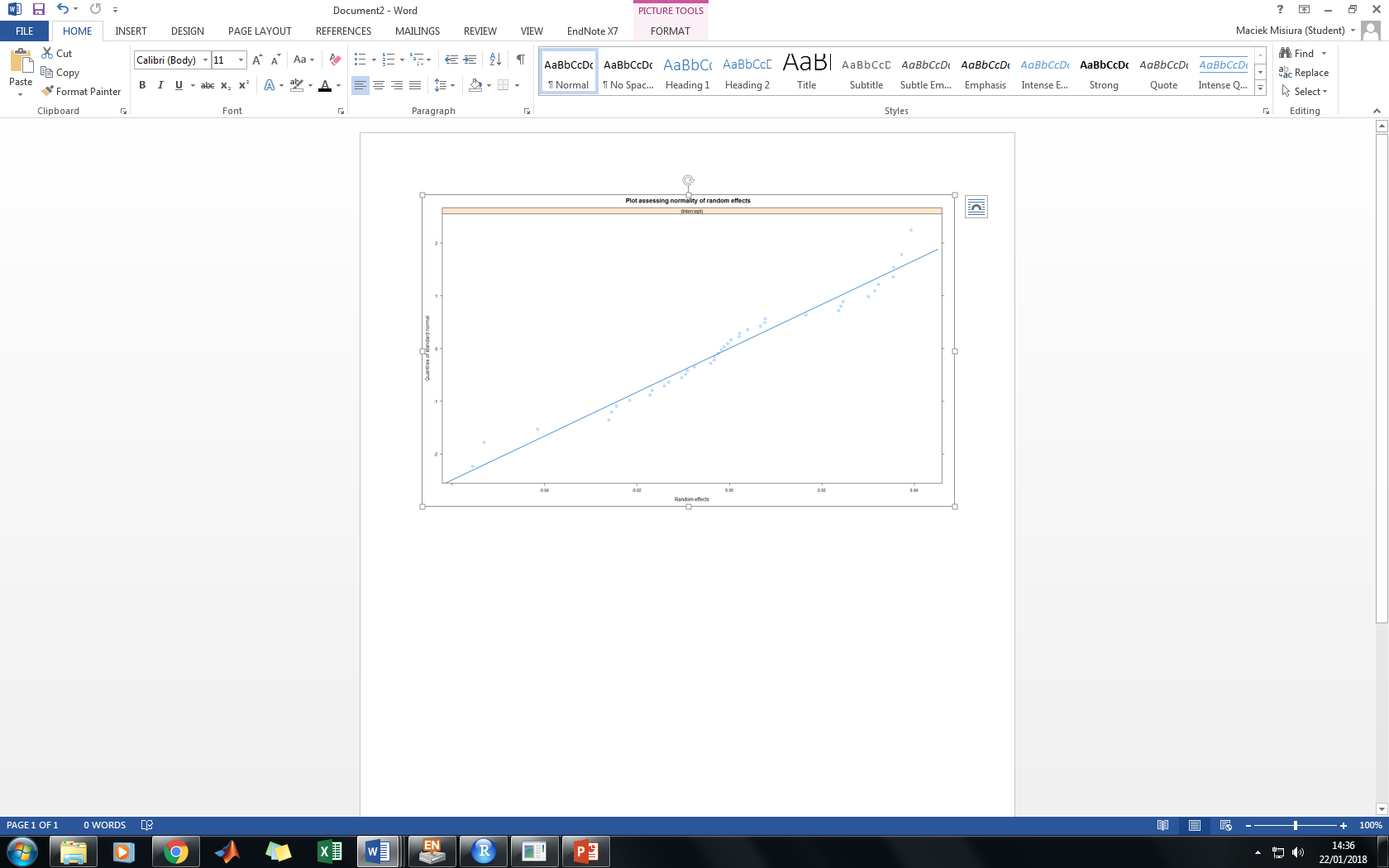


**Supplementary Material D: List of studies included in the systematic review and meta-analysis**

1. Jolliff JS, Mahan DC (2013) Effect of dietary calcium and phosphorus levels on the total tract digestibility of innate and supplemental organic and inorganic microminerals in a corn-soybean meal based diet of grower pigs. Journal of animal science 91, 2775-2783.
2. Gutzwiller A, Hess HD, Adam A et al. (2011) Effects of a reduced calcium, phosphorus and protein intake and of benzoic acid on calcium and phosphorus metabolism of growing pigs. Animal feed science and technology 168, 113-121.
3. Stein HH, Adeola O, Cromwell GL et al. (2011) Concentration of dietary calcium supplied by calcium carbonate does not affect the apparent total tract digestibility of calcium, but decreases digestibility of phosphorus by growing pigs. Journal of Animal Science 89, 2139-2144.
4. Fa Liao S, Sauer WC, Htoo JK et al. (2007) Effect of phytase supplementation to a high-and a low-phytate diet for growing pigs on the utilization of phosphorus and calcium. Interciencia 32.
5. Fan MZ, Li TJ, Yin YL et al. (2005) Effect of phytase supplementation with two levels of phosphorus diets on ileal and faecal digestibilities of nutrients and phosphorus, calcium, nitrogen and energy balances in growing pigs. Animal Science 81, 67-75.
6. Carter SD, Cromwell GL, Colombo G et al. (1999) Effects of porcine sometotropin on calcium and phosphorus balance and markers of bone metabolism in finishing pigs. Journal of animal science 77, 2163-2171.
7. Li D, Che XR, Wang YQ et al. (1999) The effect of calcium level on microbial phytase activity and nutrient balance in swine. Asian Australasian Journal of Animal Sciences 12, 197-202.
8. Meidinger RG, Ajakaiye A, Fan MZ et al. (2013) Digestive utilization of phosphorus from plant-based diets in the Cassie line of transgenic Yorkshire pigs that secrete phytase in the saliva. Journal of animal science 91, 1307-1320.
9. Poulsen HD, Carlson D, Nørgaard JV et al. (2010) Phosphorus digestibility is highly influenced by phytase but slightly by calcium in growing pigs. Livestock Science 134, 100-102.
10. Nørgaard JV, Fernández JA, Eriksen J et al. (2010) Urine acidification and mineral metabolism in growing pigs fed diets supplemented with dietary methionine and benzoic acid. Livestock Science 134, 113-115.
11. Stein HH, Kadzere CT, Kim SW et al. (2008) Influence of dietary phosphorus concentration on the digestibility of phosphorus in monocalcium phosphate by growing pigs. Journal of animal science 86, 1861-1867.
12. Pointillart A, Colin C, Lacroix HC et al. (1995) Mineral bioavailability and bone mineral contents in pigs given calcium carbonate postprandially. Bone 17, 357-362.
13. O’Doherty JV, Gahan DA, O’Shea C et al. (2010) Effects of phytase and 25-hydroxyvitamin D 3 inclusions on the performance, mineral balance and bone parameters of grower–finisher pigs fed low-phosphorus diets. animal 4, 1634-1640.
14. Merriman LA, Stein HH (2016) Particle size of calcium carbonate does not affect apparent and standardized total tract digestibility of calcium, retention of calcium, or growth performance of growing pigs. Journal of animal science 94, 3844-3850.
15. Blaabjerg K, Thomassen AM, Poulsen HD (2015) Microbial phytase addition resulted in a greater increase in phosphorus digestibility in dry-fed compared with liquid-fed non-heat-treated wheat–barley–maize diets for pigs. animal 9, 243-248.
16. Kraler M, Schedle K, Domig KJ et al. (2014) Effects of fermented and extruded wheat bran on total tract apparent digestibility of nutrients, minerals and energy in growing pigs. Animal Feed Science and Technology 197, 121-129.
17. Létourneau-Montminy MP, Lovatto PA, Pomar C (2014) Apparent total tract digestibility of dietary calcium and phosphorus and their efficiency in bone mineral retention are affected by body mineral status in growing pigs. Journal of animal science 92, 3914-3924.
18. Liu Y, Ma YL, Zhao JM et al. (2014) Digestibility and retention of zinc, copper, manganese, iron, calcium, and phosphorus in pigs fed diets containing inorganic or organic minerals. Journal of animal science 92, 3407-3415.
19. Blaabjerg K, Nørgaard JV, Poulsen HD (2012) Effect of microbial phytase on phosphorus digestibility in non-heat-treated and heat-treated wheat–barley pig diets. Journal of animal science 90, 206-208.
20. Jolliff JS, Mahan DC (2012) Effect of dietary inulin and phytase on mineral digestibility and tissue retention in weanling and growing swine. Journal of animal science 90, 3012-3022.
21. Almeida FN, Stein HH (2010) Performance and phosphorus balance of pigs fed diets formulated on the basis of values for standardized total tract digestibility of phosphorus. Journal of animal science 88, 2968-2977.
22. Sauer W, Cervantes M, Yanez J et al. (2009) Effect of dietary inclusion of benzoic acid on mineral balance in growing pigs. Livestock Science 122, 162-168.
23. Pomar C, Gagne F, Matte JJ et al. (2008) The effect of microbial phytase on true and apparent ileal amino acid digestibilities in growing-finishing pigs. Journal of animal science 86, 1598-1608.
24. Salobir J, Kostanjevec B, Štruklec M et al. (2005) Tannins reduce protein but not phosphorus utilization of diet with added phytase in pigs. Journal of Animal and Feed Sciences 14.
25. Sauer WC, Cervantes M, He JM et al. (2003) Effect of phytase supplementation to barley-canola meal and barley-soybean meal diets on phosphorus and calcium balance in growing pigs. Interciencia 28.
26. Veum TL, Ledoux DR, Raboy V et al. (2001) Low-phytic acid corn improves nutrient utilization for growing pigs. Journal of animal science 79, 2873-2880.
27. Veum TL, Ledoux DR, Bollinger DW et al. (2002) Low-phytic acid barley improves calcium and phosphorus utilization and growth performance in growing pigs. Journal of animal science 80, 2663-2670.
28. Larsen T, Skoglund E, Sandberg A-S et al. (1999) Soaking and pelleting of pig diets alters the apparent absorption and retention of minerals. Canadian Journal of Animal Science 79, 477-483.
29. Han YM, Yang F, Zhou AG et al. (1997) Supplemental phytases of microbial and cereal sources improve dietary phytate phosphorus utilization by pigs from weaning through finishing. Journal of Animal Science 75, 1017-1025.
30. Näsi JM, Helander EH, Partanen KH (1995) Availability for growing pigs of minerals and protein of a high phytate barley-rapeseed meal diet treated with Aspergillus niger phytase or soaked with whey. Animal Feed Science and Technology 56, 83-98.
31. Jang YD, Wilcock P, Boyd RD et al. (2016) Effect of xylanase supplementation of a phytase-supplemented diet on apparent total tract digestibility in pigs. Journal of Animal Science 94, 260-263.
32. González-Vega JC, Liu Y, McCann JC et al. (2016) Requirement for digestible calcium by eleven-to twenty-five–kilogram pigs as determined by growth performance, bone ash concentration, calcium and phosphorus balances, and expression of genes involved in transport of calcium in intestinal and kidney cells. Journal of animal science 94, 3321-3334.
33. Adhikari PA, Heo JM, Nyachoti CM (2015) True and standardized total tract phosphorus digestibility in canola meals from black and yellow fed to growing pigs. Journal of animal science 93, 209-216.
34. Nørgaard JV, Højberg O, Sørensen KU et al. (2014) The effect of long-term acidifying feeding on digesta organic acids, mineral balance, and bone mineralization in growing pigs. Animal Feed Science and Technology 195, 58-66.
35. Liao SF, Monegue JS, Lindemann MD et al. (2011) Dietary supplementation of boron differentially alters expression of borate transporter (NaBCl) mRNA by jejunum and kidney of growing pigs. Biological trace element research 143, 901-912.
36. Korniewicz D, Hoffmann J, Korniewicz A et al. (2010) Effect of new feed phosphate on balance and apparent absorption of calcium and phosphorus in fattening pigs. Ann Anim Sci 10, 459-466.
37. Hu Q, Yang L, Fang J et al. (2010) Estimating an optimal ratio of true digestible Ca: P in corn-roughsoybean meal-based diets for 20-50 kg growing pigs. J Food, Agric & Environ 8, 556-562.
38. Rideout TC, Fan MZ (2004) Nutrient utilisation in response to dietary supplementation of chicory inulin in growing pigs. Journal of the Science of Food and Agriculture 84, 1005-1012.
39. Nortey TN, Patience JF, Simmins PH et al. (2007) Effects of individual or combined xylanase and phytase supplementation on energy, amino acid, and phosphorus digestibility and growth performance of grower pigs fed wheat-based diets containing wheat millrun. Journal of animal science 85, 1432-1443.
40. Adeola O (2017) Ca absorption and retention data in growing and finishing pigs. Unpublished data.: Purdue University.
41. **Supplementary Material E: Sensitivity analysis: linear mixed effects regression vs linear regression with cluster robust variances**

- **Ca absorption model (linear mixed effects regression): model summary**

**Table A.** Parameter estimates, standard errors and significance levels of the main significant fixed effects and their two-way interactions in the final LMER model for Ca absorption*.*

| **Variable** | **Estimate (β)** | **Std Error (SE)** | **P-value (p)** |
| --- | --- | --- | --- |
| Intercept | -0.00159 | 0.00668 | < 0.001 |
| TCa | 0.596 | 0.0293 | < 0.001 |
| PP | -0.253 | 0.0574 | < 0.05 |
| NPP | 0.106 | 0.0524 | < 0.001 |
| ExPhyt | 0.00172 | 0.000305 | < 0.001 |
| TCa x ExPhyt | -0.00693 | 0.00101 | < 0.001 |
| TCa x PP | - | - | n.s. |
| TCa x NPP | - | - | n.s. |
| PP x ExPhyt | 0.0121 | 0.00312 | < 0.001 |
| PP x NPP | - | - | n.s. |
| NPP x ExPhyt | - | - | n.s |
| Pig genotype | - | - | n.s |
| Pig gender | - | - | n.s |

**TCa** – total Ca intake; **TP** – total P intake; **PP** – phytate-P intake; **NPP** – non-phytate-P intake; **ExPhyt** – exogenous phytase intake.

- **Ca absorption model (linear regression with cluster robust variances): model summary**

**Table B.** Parameter estimates, standard errors and significance levels of the main significant fixed effects and their two-way interactions in the final cluster regression model for Ca absorption*.*

| **Variable** | **Estimate (β)** | **Std Error (SE)** | **P-value (p)** |
| --- | --- | --- | --- |
| Intercept | 0.0136 | 0.00808 | n.s |
| TCa | 0.564 | 0.0380 | < 0.001 |
| PP | -0.247 | 0.0547 | < 0.001 |
| NPP | 0.121 | 0.0875 | < 0.05 |
| ExPhyt | 0.00115 | 0.000267 | < 0.001 |
| TCa x ExPhyt | -0.00628 | 0.00174 | < 0.001 |
| TCa x PP | - | - | n.s. |
| TCa x NPP | - | - | n.s. |
| PP x ExPhyt | 0.0103 | 0.00495 | < 0.05 |
| PP x NPP | - | - | n.s. |
| NPP x ExPhyt | - | - | n.s |
| Pig genotype | - | - | n.s |
| Pig gender | - | - | n.s |

**TCa** – total Ca intake; **TP** – total P intake; **PP** – phytate-P intake; **NPP** – non-phytate-P intake; **ExPhyt** – exogenous phytase intake.

- **Ca retention model linear mixed effects regression): model summary**

**Table C.** Parameter estimates, standard errors and significance levels of the main significant fixed effects and their two-way interactions in the final LMER model for Ca retention*.*

| **Variable** | **Estimate (β)** | **Std Error (SE)** | **P-value (p)** |
| --- | --- | --- | --- |
| Intercept | -0.0179 | 0.00954 | < 0.001 |
| TCa | 0.430 | 0.0298 | < 0.001 |
| PP | -0.0188 | 0.0814558 | < 0.01 |
| NPP | 0.313 | 0.0524 | < 0.001 |
| ExPhyt | 0.00157 | 0.000298 | < 0.001 |
| TCa x ExPhyt | -0.00431 | 0.000870 | < 0.001 |
| TCa x PP | - | - | n.s. |
| TCa x NPP | - | - | n.s. |
| PP x ExPhyt | 0.00553 | 0.002584 | < 0.05 |
| PP x NPP | - | - | n.s. |
| NPP x ExPhyt | - | - | n.s |
| Pig genotype | - | - | n.s |
| Pig gender | - | - | n.s |

**TCa** – total Ca intake; **TP** – total P intake; **PP** – phytate-P intake; **NPP** – non-phytate-P intake; **ExPhyt** – exogenous phytase intake.

- **Ca retention model (linear regression with cluster robust variances): model summary**

**Table D.** Parameter estimates, standard errors and significance levels of the main significant fixed effects and their two-way interactions in the final cluster regression model for Ca retention*.*

| **Variable** | **Estimate (β)** | **Std Error (SE)** | **P-value (p)** |
| --- | --- | --- | --- |
| Intercept | -0.0151 | 0.0136 | n.s |
| TCa | 0.433 | 0.0486 | < 0.001 |
| PP | -0.0503 | 0.0910 | n.s |
| NPP | 0.213 | 0.107 | < 0.05 |
| ExPhyt | 0.00122 | 0.000356 | < 0.001 |
| TCa x ExPhyt | -0.00416 | 0.00183 | < 0.05 |
| TCa x PP | - | - | n.s. |
| TCa x NPP | - | - | n.s. |
| PP x ExPhyt | 0.00478 | 0.00298 | < 0.05 |
| PP x NPP | - | - | n.s. |
| NPP x ExPhyt | - | - | n.s |
| Pig genotype | - | - | n.s |
| Pig gender | - | - | n.s |

**TCa** – total Ca intake; **TP** – total P intake; **PP** – phytate-P intake; **NPP** – non-phytate-P intake; **ExPhyt** – exogenous phytase intake.
